# Fibrocytes and the pathogenesis of diffuse parenchymal lung disease

**DOI:** 10.1186/1755-1536-5-S1-S22

**Published:** 2012-06-06

**Authors:** Borna Mehrad, Robert M Strieter

**Affiliations:** 1 Division of Pulmonary and Critical Care Medicine, Department of Medicine, University of Virginia, Charlottesville, Virginia, USA

## Abstract

Fibrosis is fundamental to the pathogenesis of many chronic lung diseases, including some lung infections, airway diseases such as bronchiectasis and asthma, and most of the diffuse parenchymal lung diseases. Idiopathic pulmonary fibrosis, the prototypical fibrotic lung disease, is amongst the most common diffuse parenchymal lung diseases and is characterized by progressive decline in lung function and premature death from respiratory failure. The clinical management of patients with this illness is hampered by our current inability to predict clinical deterioration and lack of an effective therapy. Fibrocytes are a population of bone marrow-derived circulating progenitor cells that home to injured tissues and differentiate into fibroblasts and myofibroblasts, thus contributing to scar formation. We summarize the evidence supporting the role of these cells in the pathogenesis of fibrotic lung diseases.

## Fibrosis and lung disease

Fibrosis and tissue remodeling are critical components of many diseases of the lungs. The most common categories of lung disease, in order of frequency, are infections, airway diseases resulting from environmental exposures and bronchogenic carcinoma. Tissue remodeling and deposition of extracellular matrix is detectable in all of these illnesses and is fundamental to the pathogenesis of the first two: Focal lung fibrosis is beneficial to the host in the most common lung infections, namely infections caused by mycobacteria and endemic fungi, by sequestering microorganisms that cannot be eliminated by the host. Fibrosis is also integral to development and progression inflammatory airway diseases, including asthma, chronic bronchitis and bronchiectasis, where histological development of airway remodeling correlates with irreversible loss of lung function. Deposition of extracelluar matrix is also detectable in many cancers, although its relevance to pathogenesis is less clear.

The archetypal fibrotic lung diseases are a less common group of illnesses referred to as diffuse parenchymal lung diseases -- or less formally, as interstitial lung diseases. This category encompasses a heterogeneous group of diseases defined by chronic and multifocal inflammation and fibrosis of the parenchyma, with involvement of alveolar, interstitial and vascular spaces of the lungs. Diffuse parenchymal lung diseases include several fibrotic illnesses of known cause: these are pathological responses to inhaled inorganic dusts (pneumoconioses), hypersensitivity responses to normally innocuous organic antigens (extrinsic allergic alveolitis or hypersensitivity pneumonitis), lung fibrosis in the context of multi-system autoimmune diseases (connective tissue disease-associated interstitial lung diseases), and lung fibrosis as a response to drugs or radiation. Diffuse parenchymal lung diseases also encompass a large number of lung diseases of unknown cause -- the latter group includes many well-defined clinical entities, such as sarcoidosis; it also encompasses a group of illnesses, referred to as idiopathic interstitial pneumonias, that have historically been difficult to define clinically, and whose identification necessitates the collaboration of expert pulmonary physicians, radiologists and pathologists.

## Idiopathic pulmonary fibrosis

The idiopathic interstitial pneumonias encompass several histopathologic patterns, and include idiopathic pulmonary fibrosis (IPF). IPF is the single most common of all diffuse parenchymal lung diseases and is a remarkable disease in 2 respects: first, it is an illness characterized by progressive fibrosis that culminates in death from respiratory failure a median of 3 years after diagnosis [1,2]; as such, it is the quintessential disease of pathological fibrosis. Second, no treatment short of lung transplantation has been shown to affect the natural history of IPF: small studies that showed potential benefit for immunosuppressive regimens, in retrospect included patients that had diffuse parenchymal lung diseases other than IPF and were complicated by substantial drug toxicities; meta-analyses do not support the use of corticosteroids or non-steroid immunosuppressive agents in this disease [3,4] and the utility of prednisone and azathioprine, a commonly used regimen, was again disproved recently [5]. Clinical trials of interferon gamma-1b, imatinib, etanercept, sildenafil and bosentan have yielded negative results [6-12]. The data regarding benefit of pirfenidone is contradictory [12-16] leading to approval of its use in Europe and Japan but not the United States; an expert panel has recommended against its use based on the observation that any beneficial effect of pirfenidone is expected to be outweighed by significant adverse effects [17]. The value of oral N-acetyl cysteine as monotherapy has not been established and is under study [5]. A recent study of an inhibitor of a tyrosine kinase downstream of multiple growth factor receptors did not affect the primary outcome, decline in FVC, but showed a reduction in incidence of acute exacerbation at the highest dose tested, a potentially important finding if it is reproducible [18]. In summary, due to its relative frequency and lack of proven therapeutic options, there is a pressing clinical need for new approaches to the management of IPF.

The lung histologic pattern in IPF is termed usual interstitial pneumonia (UIP). The UIP histologic pattern is not unique to IPF and also occurs in several diseases in which the injurious agent is known, such as pneumoconioses, chronic hypersensitivity pneumonitis and some cases of the lung diseases due to connective tissue disorders. The UIP pattern consists of normal areas of lung juxtaposed with areas of leukocyte infiltration and other areas with advanced fibrosis in a given low-power field [17,19]. This variegated pattern has led to the hypothesis of temporal heterogeneity. This hypothesis states that UIP is the consequence of multiple episodes of inflammation and fibrosis in response to multiple insults over time, and not the response to a single insult [17]. Another histological feature of UIP is the presence of fibroblastic foci, areas of polyclonal fibroblast and myofibroblast aggregation and site of deposition of new collagen, thought to represent areas of fibroblast proliferation and differentiation, and are located at the edges of areas of dense fibrosis [17,20].

Given their pivotal role in the generation of the extracellular matrix, fibroblasts and myofibroblasts are considered the primary effector cells in the evolution of pulmonary fibrosis, and their cellular source is thus a critical question in the pathogenesis of fibrotic lung diseases. Currently, 3 hypotheses address the origin of lung fibroblasts [21-23]: The classical concept is that tissue injury induces the activation, proliferation and differentiation of a resident fibroblast in the lung interstitial compartment into a myofibroblast that migrates into the alveolar compartment and expresses constituents of the extracellular matrix leading to lung fibrosis. The second mechanism involves injury-induced changes in the microenvironment of the epithelium or endothelium, inducing their transition to a mesenchymal phenotype and subsequently contributes to fibroproliferation. The third mechanism pertains to a circulating bone marrow-derived progenitor cell, the fibrocyte, that can home to sites of lung injury, differentiate into fibroblasts and myofibroblasts, proliferate, and contribute to the generation of extracellular matrix.

## Relevance of fibrocytes to lung fibrosis in animal models

Using the mouse model of intrapulmonary bleomycin administration, CD45+ Col1+ CXCR4+ fibrocytes were found to increase in the peripheral blood of animals challenged with intrapulmonary bleomycin as compared to saline, and then return to steady state levels [24]. In contrast, lung fibrocytes began to appear in the lung 2 days after bleomycin administration, peak at 8 days after bleomycin and remained elevated between days 16 and 20; this time course correlated temporally with deposition of collagen in the lungs and suggested (but did not prove) mobilization of fibrocytes from the bone marrow to the blood and subsequently to the lungs. To address this issue definitively, several investigators have stable radiation chimeric mice in which bone marrow cells express enhanced GFP on the beta-actin promoter. These works have demonstrated that in response to intra-pulmonary bleomycin or lung irradiation, fibrocytes traffic and accumulate in the lungs [25,26] and a subset differentiates into myelofibroblasts [27]. Consistent with this, when purified human fibrocytes were intravenously transferred to SCID mice that had been exposed to either bleomycin or saline, greater numbers of human CD45+ Col1+ CXCR4+ fibrocytes were observed in bleomycin-challenged lungs as compared to saline-treated controls [24].

In examining the chemokine receptor profile of circulating CD45+ Col1+ cells in mice, we have found that both in normal mice and animals challenged with bleomycin, CXCR4 is the most commonly expressed surface receptor, being present in approximately 70% of the cells [27]. In addition, CXCL12, the ligand for CXCR4, is expressed in the lungs and is induced after intrapulmonary administration of bleomycin within one day and then remains elevated for the subsequent 19 days; the dynamics of this expression therefore are consistent with a role for CXCL12 in recruiting CXCR4+ fibroytes to the lungs. Indeed, in vivo neutralization of CXCL12 resulted in reduced number of lung CD45+ Col1+ CXCR4+ fibrocytes and α-SMA-expressing myofibroblasts as well as reduced lung collagen content and attenuated pulmonary fibrosis by histologic morphometric analysis, but did not influence the number of lung neutrophils, macrophages, CD4 and CD8 T cells or NK cells [24]. Consistent with this, pharmacological antagonism of CXCR4 also results in reduced lung fibrocyte numbers and pulmonary fibrosis in response to bleomycin [28].

Work by other groups has examined the role of other mechanisms in recruitment of fibrocytes to the lungs in animal models of lung fibrosis. Using a model of intrapulmonary fluorescein isothiocyanate-induced lung fibrosis, fibrocytes were isolated from lung tissue and bronchoalveolar lavage after in vitro culture [29]. These cells expressed CXCR4, CCR5, CCR7 and CCR2 and migrated in response to CCL2 and CCL12 ligands. CCR2-deficient mice treated with intratracheal FITC were found to have lower levels of fibrocytes in the lung and less fibrosis as compared to wildtype counterparts [30], and effect that was later found to be independent of CCL2, but was attributed to another CCR2 ligand, CCL12 [29]. CCR2 is also highly expressed on cells of the mononuclear phagocyte lineage including monocyte, macrophage and dendritic cell populations, however, and reduced lung fibrosis in response to bleomycin in CCR2 knockout animals correlated with a substantial reduction in these cells as well as inflammatory cytokines in the bronchoalveolar lavage fluid [31,32]; it is therefore not clear whether the observed effect in CCR2-deficient animals is attributable to fibrocytes or other cell populations. Fibrocyte influx to the lung in the bleomycin model has also been linked to the CCL3-CCR5 chemokine axis; interestingly, this effect was associated with reduced lung expression of lung CXCL12 expression in the lungs of CCL3- and CCR5-deficient animals, suggesting that the effect of CCL3-CCR5 may be mediated via the CXCL12-CXCR4 axis [33]. Finally, in a model of pulmonary over-expression of TGF-β, the number of lung fibrocytes and the extent of lung fibrosis were attenuated by deletion of semaphorin 7a (a cell surface receptor that interacts with β1 integrin) in bone marrow-derived cells [34].

## Are fibrocytes and CXCL12/CXCR4 relevant to human interstitial lung disease?

Mouse models of lung fibrosis are helpful in identifying potential mechanisms of human disease and to test causal relationships, but differ from human diffuse parenchymal lung diseases in a number of important ways: Importantly, most animal models of lung fibrosis are caused by a single discreet exposure to an injurious agent, which results in acute lung injury and inflammation, followed by a fibrotic stage, which eventually resolved in surviving animals. In contrast, human IPF does not have an identifiable inciting event, is characterized by a relapsing and remitting course rather than a monophasic progression, and is progressive rather than resolving. In addition, lung fibrosis in mouse models of lung fibrosis does not show histological evidence of temporal heterogeneity or fibroblastic foci, the hallmarks of human UIP. Consistent with this, there are marked differences between mechanisms identified in the bleomycin-induced mouse model of pulmonary fibrosis and human disease [35].

Similar to mouse fibrocytes, CXCR4 is the most prominently expressed chemokine receptor on fresh peripheral blood human fibrocytes, being expressed by 85% of circulating CD45+ Col1+ cells; by comparison, CCR2 and CCR7 are expressed by approximately 50% and 10% of human blood fibrocytes [27]. Both CCR2 and CXCR4 expressed by fibrocytes are functional because human cells display in vitro chemotaxis towards the CXCR4 ligand, CXCL12 [27] and the CCR2 ligand CCL2 [36]. In addition, the chemokine ligand, CXCL12, was found to be extensively expressed in lung tissue from patients with the histologically diagnosed interstitial lung disease as detected by immunohistochemistry and ELISA, but not in histologically normal lungs [37]. A similar increase in plasma CXCL12 levels in patients with interstitial lung disease, supporting the concept that there is a CXCL12 gradient between the bone marrow and the blood to allow release of fibrocytes from the bone marrow in these patients. Consistent with this, there was >5-fold higher concentration of circulating CD45+ Col1+ fibrocytes in unmanipulated peripheral blood of patients with interstitial lung disease as compared to healthy controls, approximately 10% of which express αSMA, suggesting further differentiation to myofibroblast phenotype [37]. These findings have now been corroborated by 2 other groups, showing expanded fibrocyte pool in the lungs and blood of subjects with scleroderma-associated interstitial lung disease, associated with increased CXCL12 expressions in the lungs [38,39]. In addition, fibrocytes have been documented in lung biopsy specimens of subjects with IPF, where their numbers was associated with elevated plasma and bronchoalveolar fluid CXCL12 and correlated with the number of fibroblastic foci [40].

Most recently the correlation of circulating fibrocytes to prognosis of IPF patients was tested. The proportion of circulating CD45+ Col1+ cells in the buffy coat was found to be elevated in patients with stable IPF as compared to healthy controlled, and greatly increased in subjects experiencing exacerbations of IPF, in whom it returned to baseline levels in survivors who recovered from the exacerbation [41]. The ratio of circulating fibrocytes did not correlate with physiological parameters in IPF patients but was a powerful predictor of survival: subjects with <5% circulating fibrocytes has a median survival of 27 months as compared to subjects with >5% fibrocytes whose median survival was 7.5 months [41].

## Can fibrocyte CXCR4 expression be manipulated therapeutically?

As noted above, CXCR4 is the predominant chemokine receptor on human and mouse fibrocytes, and interrupting the CXCR4-CXCL12 axis in mice results in attenuation of fibrosis. Furthermore, both hypoxia and growth factors result in increase CXCR4 mRNA, CXCR4 cell surface expression, and chemotaxis to CXCL12 in human fibrocytes [27]. This augmentation can be abrogated by exposing cultured human fibrocytes to the mTOR inhibitor sirolimus in vitro [27]. In the in vivo setting, treatment of bleomycin-challenged mice with sirolimus has been shown to result in reduced absolute number of CXCR4+ fibrocytes in the blood and lungs but did not influence the basal numbers of fibrocytes in the peripheral blood or lung in mice treated with saline instead of bleomycin. Consistent with its effect on fibrocyte infiltration, sirolimus treatment resulted in an approximately 60% decrease in lung collagen deposition [27]. This result is consistent with a prior report of effectiveness of sirolimus in a rat model of pulmonary fibrosis [42], but does not exclude the possibility of effects of sirolimus that may be independent of CXCR4 expression or, indeed, fibrocytes. Given the limited therapeutic options and poor prognosis of human IPF, lack of optimal animal models that recapitulate the human disease, biological plausibility of a potential benefit for mTOR inhibition in this disease, and the clinical availability of mTOR inhibitors, a case can be made to test this drug in a pilot study in human IPF.

## Conclusions

Human diffuse parenchymal lung diseases are a heterogeneous group of illnesses characterized by various degrees of lung inflammation and fibrosis. Data from animal models, clinical studies and in vitro experiments support a role for bone marrow derived fibrocytes in the pathogenesis of these conditions. This information may prove useful in the management of these diseases in two ways: first, circulating fibrocyte concentration may be a biomarker of disease activity and prognosis, and second, fibrocyte biology represents a potential therapeutic target in these diseases.

## Competing interests

The authors declare that they have no competing interests.
